# The Plastic Interplay between Lung Regeneration Phenomena and Fibrotic Evolution: Current Challenges and Novel Therapeutic Perspectives

**DOI:** 10.3390/ijms25010547

**Published:** 2023-12-31

**Authors:** Sara Lettieri, Francesco R. Bertuccio, Lucia del Frate, Fabio Perrotta, Angelo G. Corsico, Giulia M. Stella

**Affiliations:** 1 Department of Internal Medicine and Medical Therapeutics, University of Pavia Medical School, 27100 Pavia, Italy; sara.lettieri01@libero.it (S.L.); francesco.bertuccio01@gmail.com (F.R.B.); lucia.delfrate01@universitadipavia.it (L.d.F.); angelo.corsico@unipv.it (A.G.C.); 2 Cardiothoracic and Vascular Department, Unit of Respiratory Diseases, IRCCS Policlinico San Matteo, 27100 Pavia, Italy; 3 Department of Translational Medical Science, University of Campania Luigi Vanvitelli, 80055 Naples, Italy; fabio.perrotta@unicampania.it

**Keywords:** fibrosis, regeneration, mesenchymal stem cells

## Abstract

Interstitial lung diseases (ILDs) are a heterogeneous group of pulmonary disorders characterized by variable degrees of inflammation, interstitial thickening, and fibrosis leading to distortion of the pulmonary architecture and gas exchange impairment. Among them, idiopathic pulmonary fibrosis (IPF) displays the worst prognosis. The only therapeutic options consist of the two antifibrotic drugs, pirfenidone and nintedanib, which limit fibrosis progression but do not reverse the lung damage. The shift of the pathogenetic paradigm from inflammatory disease to epithelium-derived disease has definitively established the primary role of type II alveolar cells, which lose their epithelial phenotype and acquire a mesenchymal phenotype with production of collagen and extracellular matrix (EMC) deposition. Some predisposing environmental and genetic factors (e.g., smoke, pollution, gastroesophageal reflux, variants of telomere and surfactant genes) leading to accelerated senescence set a pro-fibrogentic microenvironment and contribute to the loss of regenerative properties of type II epithelial cells in response to pathogenic noxae. This review provides a complete overview of the different pathogenetic mechanisms leading to the development of IPF. Then, we summarize the currently approved therapies and the main clinical trials ongoing. Finally, we explore the potentialities offered by agents not only interfering with the processes of fibrosis but also restoring the physiological properties of alveolar regeneration, with a particular focus on potentialities and concerns about cell therapies based on mesenchymal stem cells (MSCs), whose anti-inflammatory and immunomodulant properties have been exploited in other fibrotic diseases, such as graft versus host disease (GVHD) and COVID-19-related ARDS.

## 1. Introduction

**Figure 1:**
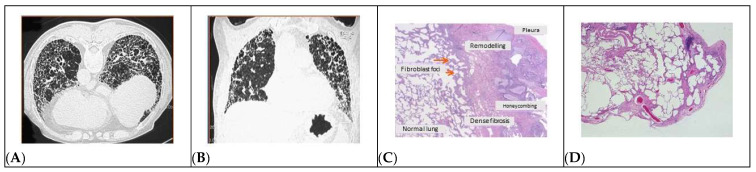
Imaging and pathological presentation of IPF. Spatial and temporal heterogeneity is a key feature of the disease detectable in both CT scans and morphologic studies. The high-resolution CT scan with the patient in prone decubitus demonstrates the presence of a structural subversion of the parenchyma with evident honeycombing associated with bronchiectasis and traction bronchiolectasis. In the multiplanar reconstruction in coronal section, an extension of the alterations to the upper lobes is observed, typical of the advanced stages of the disease. Panel (**A**), axial thoracic CT scan section showing bi-basal honeycombing alteration; panel (**B**), coronal section of the same case pointing out the peripheral distribution of the lesions. The matched histologic samples in different magnification levels shows dense fibrotic areas near healthy lung, fibroblast foci in association with subpleural cysts, and interstitial inflammation. Panel (**C**), hematoxylin and eosin stain (40×) showing remodeling areas next to active fibroblastic proliferations (fibroblast foci); panel (**D**), higher definition (100×) of the lung parenchyma carrying cystic alterations and fibroblast foci [10].

The most prevalent kind of idiopathic interstitial pneumonia is called idiopathic pulmonary fibrosis (IPF) [1,2], a chronic fibrotic illness of the lower respiratory tract that progresses relentlessly [1]. Usual interstitial pneumonia is the term used to describe the histopathologic pattern linked to the clinical diagnosis of interstitial pneumonia (UIP). UIP is also observed in chronic hypersensitivity pneumonitis, asbestosis, and other fibrotic lung disorders linked to connective tissue diseases [2]. In IPF patients, abnormal proliferation of mesenchymal cells, varying degrees of fibrosis, overproduction and disordered deposition of collagen and extracellular matrix, distortion of pulmonary architecture, and subpleural cystic airspaces (3 to 10 mm diameter) known as honeycomb cysts are the characteristic histopathologic features of UIP. Fibroblast foci (FF), a distinctive histologic hallmark of UIP, identify clusters of fibroblasts and myofibroblasts that lie in continuity with the existing fibrosis [1,3,4,5,6]. The intricate, highly linked reticulum that emerges from the fibrotic process extends from the pleura into the underlying parenchyma (Figure 1) [7]. IPF is considered a rare disease, as it affects fewer than 1 in 2000 individuals, with an incidence that, however, varies depending on the study and appears to be increasing. The incidence of the disease varies depending on age and sex. Males are the most affected and the disease is rare before the age of 50 but is present in an estimated 0.2% of people over the age of 75. IPF is clinically characterized by irreversible loss of lung function due to fibrosis, manifesting as chronic cough and progressive worsening of dyspnoea, compounded by a median survival from diagnosis of approximately 3–5 years, although it is difficult to predict the clinical course in each case, as it is very variable [1,2]. Working with animal models of pulmonary fibrosis has provided valuable insights into the mechanisms of IPF onset and progression. However, there are some differences between pulmonary fibrosis in animals and IPF in humans. Despite that, there are enough parallels between some animal models and human illness to allow for the development of several understandings and theories regarding the pathophysiology of IPF [8,9]. The aim of the present review is, thus, to summarize the state of the art regarding the knowledge of the biological basis and therapeutic options against IPF, taking into main account the role of mesenchymal stem cells as mediators of tissue remodeling and renewal.

## 2. Predisposing Factors

**Figure 2:**
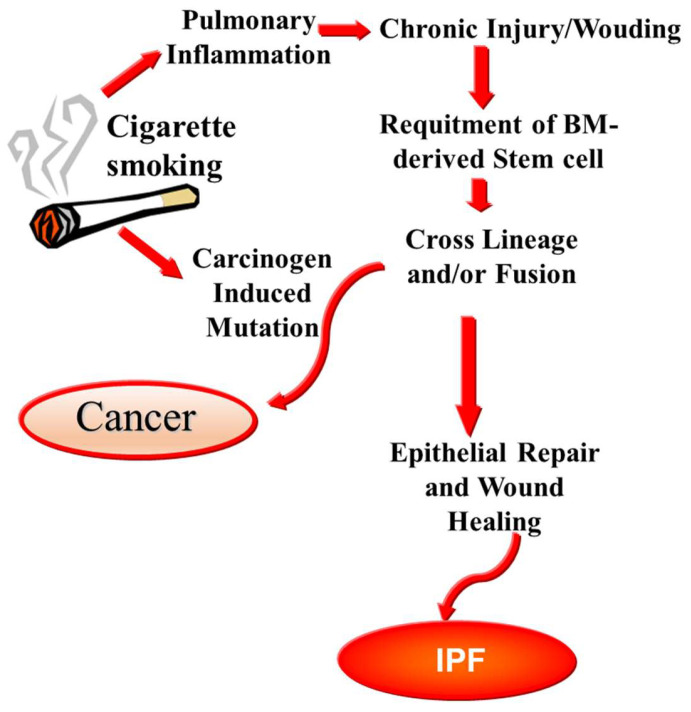
Smoking habit as risk factor for both IPF and cancer. Chronic cigarette smoking results in lung inflammation and epithelial damage that activates a chronic wound repair programme. Bone marrow (BM)-derived stem cells can respond to epithelial damage and contribute to its repair. They ultimately can differentiate into epithelial cell through directly crossing the lineage or fusion with lung epithelial cells. The aberrant proliferative phenotype is a key feature of IPF; when carcinogens in cigarette smoke induce the occurrence of genetic mutations, malignant transformation of epithelial cells can occur as well (for a review, see [13,14,15]).

The specific variables responsible for the histopathologic processes seen in IPF remain largely unexplored. The development of IPF is linked to several heterogenous risk factors, including drug use, genetic predisposition, environmental contaminants, chronic aspiration, viral infection, and cigarette smoking (Figure 2). Nevertheless, none of these variables can fully account for the profound remodeling and progressive character of IPF. In susceptible people, the disease process of IPF may be initiated by nonspecific damage to the pulmonary parenchyma and epithelium [11,12].

### 2.1. Genetic Predisposition 

Genome-wide association studies and reports of families with several sick members (familial pulmonary fibrosis) corroborate a genetic predisposition to pulmonary fibrosis. Nevertheless, it is still uncertain which specific genetic and host susceptibility factor(s) govern the phenotypic expression and clinical presentation of sporadic IPF [3,10]. Numerous gene variations have been discovered that could be involved in the etiology of pulmonary fibrosis. Indeed, gene variations are implicated in familial clusters of the disease and this association has led to the definition of familial interstitial pneumonia (FIP). Many already-published papers are available regarding gene variants in IPF and FIP [16,17,18]. Overall, the occurrence of genetic variants, both rare (those with minor allele frequency of <0.1%) or common (minor allele frequency of >5%), is associated to all those processes that are known to play a role in fibrogenesis, such as alteration of pneumocyte homeostasis, host defense, cell–cell barrier, and cell senescence. Several rare variants have been reported in FIP cases, such as those related to alveolar stability [*SFTPC*, *SFTPA1*, *SFTPA2*, ATP-binding cassette-type 3 (*ABCA3*), and *NAF1* as well as five genes linked to telomere biology [*TERT*, *TERC*, *DKC1* [17], *TINF2*, *RTEL1*, and PARN]. 

### 2.2. Surfactant Proteins

Lung surfactant is a surface-active complex made of phospholipids and proteins lining the alveolar epithelium whose main function is to prevent alveolar collapse during expiration. Moreover, it is well-known that it plays a crucial role in immune responses and inflammatory cascades [19,20]. In multiple IPF families, variations of surfactant proteins A2 (SP-A2) and C (SFTPC) have been found [21,22]. Heterozygosity affecting SFTPC has been associated with the occurrence of fibrosis at all ages, including pediatric forms of interstitial lung diseases (pILDs). Adenosine triphosphate binding cassette family of transporters A3 (ABCA3) gene variations, most often resulting in missense variants, small insertions, or deletions, may have an impact on how SFTPC-related IPF develops and in pILD [23,24]. In those cases, a substitution of isoleucine by threonine in codon 73 (I73T) has been reported [24]. The clinical analysis showed that each infant develped respiratory symptoms by 2 months of age and inherited the mutation from an asymptomatic parent. The disease progresses during childhood and adolescence, leading to severe respiratory failure. Until now, the only therapeutic option is lung transplantation, but gene-based therapies, such as gene replacement or editing, hold promise for the future [25]. 

### 2.3. Mucin 5B

A common variation was discovered in the promoter of the gene producing mucin 5B after a genome-wide scan on chromosome 11 revealed a risk locus for IPF (*MUC5B*) [26]. This variation was found in 34% of people with IPF, 38% of subjects with familial pulmonary fibrosis, and 9% of controls. A single-nucleotide polymorphism in the *MUC5B* promoter was found to be substantially linked to IPF in a different study, but not to lung fibrosis in sarcoidosis or systemic sclerosis [27]. MUC5B expression in lung tissue is 14 times higher in people with IPF compared with normal subjects. *MUC5B* mutations cause three times the risk of IPF compared to the total risk of 13 other susceptibility variants [28].

### 2.4. Telomerase-Related Genes 

Variants in telomerase-related genes as *TERT*, *RTEL1*, *TERC*, *DKC1*, and *TINF2* resulting in shortened telomeres have been found to be involved in approximately 25% of sporadic IPF cases and 15% of familial pulmonary fibrosis cases [29,30,31,32,33]. It is not always the case that patients with sporadic IPF and telomere shortening have distinguishable variations in their telomerase genes, and a higher percentage of lung fibroblasts from IPF patients than from controls exhibit telomerase activity induction [34]. The gene that codes for the regulator of telomere elongation helicase 1 (*RTEL1*) has nine related loss-of-function variants [35]. A DNA helicase called RTEL1 controls the stability and replication of telomeres. The telomerase gene *TERT*, the mucin gene *MUC5B*, and telomeres have all been linked to idiopathic interstitial pneumonias (IIPs) in genome-wide association studies (GWAS).

### 2.5. Other Genes 

Although a detailed analysis of GWSA results goes beyond the scope of this work, it should be underlined that additional genes contributing to a predisposition to IPF have been reported in conjunction with investigations of rare variants or gene expression in patients with lung fibrosis [36]. 

### 2.6. A-Kinase Anchoring Protein 13 

A unique association signal near A-kinase anchoring protein 13 was discovered in individuals with IPF of European descent who participated in a genome-wide association study conducted in the United Kingdom (AKAP13; rs62025270). In lung alveolar epithelium and lymphoid follicles from IPF patients, there was an increase in the expression of AKAP13 mRNA, which is linked to a higher vulnerability to IPF. Targeting the RhoA pathway inhibitors may be helpful for patients with IPF, as AKAP13 interacts with both protein kinase A and the Rho/Rac family of guanosine triphosphate binding proteins [37].

### 2.7. Kinesin Family Member 15 

Excess uncommon detrimental mutations of kinesin family member 15 (KIF15) were found in the whole-genome sequencing of 1725 individuals with familial or sporadic IPF; comparable results were seen in separate replication cohorts. The single-nucleotide polymorphism (rs74341405) that showed the strongest correlation in this investigation had an allele frequency of roughly 6–7% in IPF patients and 4% in healthy controls. KIF15 is expressed in lung epithelial cells that are reproducing, macrophages, and T cells. It is involved in spindle separation during mitosis. While replicating epithelial cells of IPF patients show lower KIF15 expression in vivo, both common and unusual mutations result in reduced KIF15 expression and reduced rates of cell proliferation in vitro. These findings suggest that decreased epithelial replicative reserve may play a role in susceptibility to IPF [38].

### 2.8. Additional Loci 

Genome-wide association studies using IPF cohorts have identified multiple additional associated loci including FAM13A (4q22), DSP (6p24), MAD1L1 (7p22), ZKSCAN1 (7q21-22), DEPTOR (8q24), OBFC1 (10q24), ATP11A (13q34), IVD and KNL1 (15q15), IL9RP3 (16p13), DPP9 (19p13), TOLLIP (11p15), MDGA2 (14q21), SPPL2C (17q21), and STMN3 (20q13), as well as chromosomal regions 7q22, 10q25, and 15q14-15 [26,39]. 

### 2.9. Inflammation 

The possibility that inflammation occurs before fibrosis develops during IPF pathogenesis has been suggested by a high number of observations. In several animal models of fibrosis, inflammation occurs before a fibrotic response develops, and suppression of the inflammatory alveolitis reduces the fibrotic response that follows [40]. Histologically, inflammatory cells such as alveolar macrophages, neutrophils, eosinophils, and lymphocytes predominate in the alveolitis of early pulmonary fibrosis in animal models. There have also been reports of elevated mast and basophil counts [41]. Because the alveolar macrophage secretes profibrotic and proinflammatory cytokines that influence mesenchymal cell proliferation and promote collagen deposition, it has been suggested that the macrophage plays a crucial role in the inflammatory pathophysiology of IPF [3,9]. However, the idea that lung inflammation is necessary to produce fibrosis in IPF is disputed for several reasons: patients with IPF typically have low levels of inflammation, and there is little proof that those with the disease at an earlier stage have more noticeable inflammatory alterations [1,5]. In lab animals, fibrosis can be generated in the absence of inflammation [8]. Furthermore, systemic glucocorticoid-administered anti-inflammatory medication has not been able to change the normal course of IPF, and certain anti-inflammatory regimens exacerbate clinical symptoms.

### 2.10. Fibroblast and Epithelial Cell Dysfunction 

There is, however, another idea that suggests that IPF has little to no inflammatory component and is instead caused by aberrant fibroblast and epithelial cell activity as well as defective epithelial–mesenchymal interactions [42]. The appearance of fibrotic foci immediately beneath regions of injured epithelium without a large infiltration of inflammatory cells histologically supports this notion [43,44,45]. The injured alveolar epithelium releases mediators that regulate fibroblast proliferation, such as transforming growth factor beta (TGF-β), connective tissue growth factor (CTGF), Sonic hedgehog protein (Shh), and prostaglandin E2 (PGE2). Overall, these mediators induce the activation of type two epithelial-to-mesenchymal transition [46]. This process acts as a source of myofibroblasts, since, under biochemical stimuli, epithelial cells de-differentiate and acquire a fibroblast phenotype by losing their properties such as apical–basal polarity and cell–cell adhesion [47,48]. On the other hand, activated fibroblasts act by injuring alveolar pneumocytes by producing angiotensin II and reactive oxygen species (ROS) [49]. 

All the mediators produced are involved in promoting the migration, proliferation, and activation of fibroblasts, their differentiation to myofibroblasts, and abnormal and abundant secretion of extracellular matrix proteins contributing to the onset of fibrosis in the lung [50]. Genetics, environmental triggers, and an imbalance of antioxidants and oxidants may all play a role in the onset and maintenance of this fibrotic response. An imbalance of cytokines produced by Th1 and Th2 cells may also be significant [51,52,53].

## 3. Progression to Fibrosis 

The progressive nature of the fibrotic process is another defining trait of IPF. After starting at the alveolar epithelial barrier, damage to the basement membrane and epithelial cells causes intricate interactions between cells and cytokines, which spread the fibrotic process to the alveolar walls, alveolar lumen, and finally, nearby lung parenchyma. A result of epithelial damage early on is the formation of an intra-alveolar exudate. Alveolar collapse resulting in the apposition of the denuded alveolar walls and loss of surfactant is caused by the organization of the intra-alveolar exudate [54]. It seems that the formation of intraluminal fibrosis requires damage to both the basement membrane and the epithelium. Type I epithelial cells predominate in the alveolar epithelium of a normal lung, with a comparatively modest proportion of type II epithelial cells. Type II cells multiply and develop into type I cells after injury, and they are often in charge of the re-epithelialization of damaged alveoli. In IPF patients, these cells do not, however, seem to re-epithelialize the alveolar gap [55]. This can be the result of the basement membrane’s ongoing irregularities [39]. Mesenchymal cells can then migrate from the interstitium to the wounded lung’s alveolar sections because of these anomalies [35]. It seems that mesenchymal cells’ excessive collagen deposition stops the compressed airways from expanding again. The progression of intra-alveolar exudates, fibrosis, and remodeling may be caused by recurring or chronic injury to the alveolar epithelium, according to the pattern of these alterations. Another possibility is that fibroblasts are permanently impacted by an initial damage that results in the release of cytokines. Transforming growth factor-beta1 (TGF-beta1), for instance, was released from alveolar epithelial cells for four days in an experimental model of IPF. This resulted in the induction of fibroblast growth factor (FGF)-2, which was then sequestered in the fibroblast matrix and caused an altered fibroblast phenotype with prolonged proliferation [56].

## 4. Mechanisms of Fibrosis

The exact cause of evolution towards fibrosis in IPF is still unknown. One theory is that growth factors secreted by the injured epithelial cells attract fibroblasts that eventually differentiate into myofibroblasts, and that numerous microinjuries to alveolar epithelial cells create a fibrotic environment. Alpha-smooth muscle’s actin expression is a marker for myofibroblasts, which are cells that exhibit characteristics of both smooth muscle and fibroblasts (SMA) [40]. Collagen is secreted by myofibroblasts once they are recruited to the lungs or differentiate from existing fibroblasts. An imbalance between interstitial collagenases and their tissue inhibitors causes collagen to build up [33]. Although it appears that fibrosis must begin with repeated microinjuries to the alveolar wall and distal airways, the precise extrinsic elements that may cause epithelial injury are still unclear. It is possible that microaspiration of acid into the distal lungs could be one cause of recurrent insults to the epithelial barrier, altered epithelial–mesenchymal interactions, and subsequent fibrosis in genetically predisposed individuals, as nearly 90% of patients with IPF have physiologically detectable gastroesophageal reflux, often in the absence of symptoms. If there is a link between gastroesophageal reflux and IPF, more research is needed to find it [57].

### 4.1. Altered Re-Epithelialization 

A substantial amount of data points out the importance of alveolar epithelial cells (AEC) in the etiology of IPF [34]. Studies using electron microscopes show that resident mesenchymal cells are situated in the gap between the capillary endothelium and the alveolar epithelium [58]. The regulation of cell proliferation and connective tissue synthesis by fibrogenic cytokines released from epithelial cells may be facilitated by the close proximity of mesenchymal cells to epithelial cells. According to the present theory, the alveolar epithelial, subepithelial, and adjacent endothelial basement membranes are injured by recurrent subclinical lung damage [34]. This injury allows additional cells and cytokines, as well as cells of the mesenchymal lineage, to enter the alveoli. Eventually, a highly active, proliferative, and contractile fibroblast phenotype emerges because of an intricately interconnected mechanism that is still poorly understood. Type I epithelial cells are lost in IPF while type II cells proliferate; type II cells do not differentiate into type I AECs by orderly re-epithelialization. A portion of this abnormal re-epithelialization could be attributed to an abnormal stimulation of the Wnt signalling pathway after lung damage [43]. The Wnt proteins stop beta-catenin from being phosphorylated by glycogen synthase kinase 3b (GSK3b), preventing it from moving to the nucleus and activating the transcription factors known as lymphoid enhancing factor/T-cell factor (LEF/TCF); this is thought to promote type II cell proliferation while suppressing differentiation, which in turn causes divergent epithelial regeneration at bronchoalveolar junctions and may ultimately result in epithelial–mesenchymal transition [59]. Reduced alveolar epithelium regeneration ability is another potential anomaly in IPF patients’ alveolar epithelium. Age-related or hereditary factors may contribute to the reduced regeneration in certain IPF patients. Thus, a subgroup of genetically predisposed people who may see a decline in epithelial regeneration during aging could comprise IPF patients. This theory is consistent with the finding that IPF incidence and prevalence rise with age [60]. A further well-known anomaly of AECs in individuals with IPF could be the excessive synthesis and discharge of growth factors and fibrogenic cytokines.

### 4.2. Cytokines, Growth Factors, and Other Molecules 

It is obvious that the development of the fibrotic response in IPF depends on the interaction of growth factors, cytokines, and other mediators with lung resident cells. A variety of cytokines and growth factors are produced by resident epithelial, fibroblastic, and endothelial cells in the lung, which promote fibroblast proliferation and matrix formation. Fibrosis is thought to develop as a result of an imbalance between several categories of molecules, such as angiogenic and angiostatic molecules, proinflammatory and antiinflammatory cytokines, fibrogenic and antifibrogenic polypeptides, and oxidants–antioxidants, after epithelial damage. Increased levels of transforming growth factor-beta (TGF-beta), including the active form TGF-beta1, are found in BAL from IPF patients. Because of its capacity to upregulate connective tissue synthesis, downregulate connective tissue proteases, and enhance the inhibitors of connective tissue proteases, TGF-beta1 is one of the most potent regulators of connective tissue production. In addition, TGF-beta1 can trigger several cytokines and growth factors involved in fibrosis, including interleukins, platelet-derived growth factor (PDGF), fibroblast growth factor (FGF-2), connective tissue growth factor (CTGF), and insulin-like growth factor (IGF) (ILs) [61,62,63]. As a downstream mediator of TGF-beta1, CTGF (connective tissue growth factor) affects the production of connective tissue by fibroblasts and serves as a mitogen for cells either on its own or in conjunction with other mediators. In BAL, CTGF expression is elevated in IPF patients [48,64]. Tumor necrosis factor-alpha is another cytokine linked to IPF (TNF-alpha). Although TNF-alpha expression is elevated in IPF, its precise function is unknown. TNF-alpha can promote fibroblast proliferation, trigger collagen synthesis, and boost the synthesis of TGF-beta1 and other peptide mediators. People who have polymorphisms in the *TNF-alpha* gene are more likely to develop IPF [65,66,67]. Fibrotic cytokines are overexpressed as well. More and more research is ongoing on the potential impact that chemokines such as monocyte chemoattractant protein 1 (MCP-1) may have in drawing in IPF cells. Fibrogenic molecules include TGF-beta, CTGF, IL-4, IL-13, FGF-2, IGF-1, PDGF, and GM-CSF; anti-fibrogenic molecules include IFN-gamma, IL-1, IL-10, IL-12, and IL-17. The transcription factor activator protein (AP)-1 and the fos-related protein Fra-2 appear to have played a role in the evolution of fibrosis [68]. Gene expression patterns have been used to identify additional proteins that may reflect the disease activity of IPF or contribute to its etiology. For instance, genes such as matrix metalloprotein (MMP)-7, MMP-1, surfactant protein A1, cyclin A2 (CCNA2), and alpha-defensins are overexpressed in lung tissue and blood from IPF patients. Moreover, elevated MMP-7 and MMP-1 have been seen in the peripheral blood of IPF patients, and these protein levels may be correlated with disease activity [69,70,71].

### 4.3. Fibrotic Foci 

It is commonly acknowledged that FF are the typical histological hallmark of UIP. Myofibroblasts and fibroblasts appear to assemble into fibrotic foci that occur prior to the onset of end-stage fibrosis after epithelial damage induces fibroblast activity. These FF, which are composed of clusters of actively growing fibroblasts and myofibroblasts, are located next to sites of injury to basement membranes and epithelial cells [33]. Fibroblast apoptosis inhibition may control fibroblast persistence and the development of fibroblast foci. For instance, a 50 kDa protein called pigment epithelium-derived factor (PEDF) was found to be produced in the fibroblasts of IPF lungs, suggesting a potential function for it in both fibroblast maintenance and apoptosis [72]. Under the influence of transforming growth factor beta (TGF-beta), fibroblasts develop into myofibroblasts, which leads to the expression of alpha smooth muscle actin (SMA), enhanced collagen synthesis, and decreased synthesis of tissue inhibitor of metalloproteinase 2 (TIMP-2) [73,74]. Proliferating myofibroblasts that are actively generating collagen can be used to identify fibrotic foci [58].

### 4.4. Mesenchymal Cells 

At the location of fibrotic foci, smooth muscle cells, myofibroblasts, fibroblasts, pericytes, and undifferentiated cells comprise the mesenchymal cell population. Four possible sources of fibroblasts may be identified at these foci: circulating fibrocytes, epithelial cells, perivascular adventitial and peribronchiolar fibroblasts, and bone marrow-derived marrow stem cells. The majority of these cells are found inside alveolar walls [75]. Derived from fibroblasts and epithelial cells, myofibroblasts are seen in wound granulation tissue and possess morphologic characteristics shared by both fibroblasts and smooth muscle cells. Myofibroblasts and contractile interstitial cells (CICs) are found mostly in the alveolar walls and have similar ultrastructural characteristics. Patients with IPF have higher concentrations of these cell types than in healthy lung tissue. According to one study, the fraction of parenchymal cells with mesenchymal origin was 1.8 times higher in IPF lungs than in normal lungs, and these cells multiplied more quickly in the IPF lung.

### 4.5. Fibroblast Subtypes 

When compared to fibroblasts isolated from normal lungs, the behavior of fibroblast subpopulations in IPF is different. These variations include different cell-mediated responses, unique gene and cell surface marker expression, and altered proliferative capability [38]. It is currently unknown whether these differences are the result of extended exposure to an inflammatory environment or whether they arise as primary phenomena. These modifications seem to have a significant role in the fibroblast’s transformation from a largely passive cell to a key player in the pathophysiology of IPF. The fibroblast-like cell is the primary target-effector cell that seems to control the fibrotic response in IPF, regardless of the phenotypic variations. Lung fibroblast subsets have been divided based on the expression of membrane proteins, in addition to the finding of discrete lung mesenchymal cell populations based on the expression of protein microfilaments. These cell surface protein markers include the complement 1q (C1q) receptor and the thymocyte 1 antigen (Thy 1), which exhibit clear variations in collagen deposition, shape, and proliferation rate [76,77]. Since mRNA is expressed in large quantities in HF fibroblasts and just slightly in LF cells, extraction of mRNA provided more conclusive proof that fibroblast subtypes have been identified at the gene level [78].

### 4.6. Circulating Fibrocytes 

Circulating fibrocytes originate from the hematopoietic lineage. These are identified by the expression of type I collagen (Col1), CD11b, CD13, CD34, CR45RO, MHCII, and CD86. They make up 0.1 to 0.5% of the circulating leukocyte population. It is thought that circulating fibrocytes respond to chemokines CCL21 and CXC chemokine ligand 12 by binding to CCR7 and CXCR4 receptors in damaged lung tissue (CXCL12). The ability of the fibrocytes to express connective tissue proteins and differentiate into myofibroblasts when cultured raises the possibility that fibrocytes in IPF may do the same [79,80].

### 4.7. Fibroblast Proliferation 

According to in vitro examination, the most common feature in fibroblasts isolated from IPF lungs is their increased capacity for proliferation [81,82]. Furthermore, they have a reduced ability to synthesize prostaglandin E2 (PGE2), are comparatively resistant to the antiproliferative action of PGE2, and do not increase their inducible cyclooxygenase activity in response to a range of agonists [83,84]. Acute interstitial pneumonia has been observed to exhibit enhanced tritiated thymidine uptake, a reflection of DNA replication. This finding suggests that the proliferation of local mesenchymal cell populations is the cause of increases in fibroblast-like cell counts in fibrotic lesions [85]. The pathophysiology of fibroproliferative lung lesions is supported by the participation of local mesenchymal cell proliferation, as demonstrated by a time course analysis of paraffin-embedded lung tissue slices from rats subjected to bleomycin [86].

### 4.8. Stem-like Phenotype and Properties of Myofibroblasts 

Compared to fibroblast-like cells, myofibroblasts generate much more interstitial collagen. There are two primary sources of myofibroblasts in IPF. It is thought that mediators like TGF-beta cause fibroblasts to develop into myofibroblasts. Furthermore, as shown in a renal model of fibrosis, some fibroblasts may develop from the mesenchymal transition of nearby epithelial cells [87,88]. The transdifferentiation of epithelial cells by epithelial mesenchymal transition (EMT) represents another potential source of myofibroblasts [73,89]. It has long been established that EMT, a particular kind of metaplasia, aids in the growth of malignancies. The enhancement of epidermal growth factor (EGF), fibroblast growth factor (FGF), and hepatocyte growth factor (HGF) is also facilitated by the transcription factors Smads, Slug, Snail, Scatter, lymphoid enhancing factor-1, and beta-catenin. The MET oncogene, encoding the hepatocyte growth factor (HGF) receptor, drives invasive growth, a genetic programme largely overlapping with the epithelial-to-mesenchymal transition and governing physiological and pathological processes such as tissue development and regeneration, as well as cancer. During cancer progression, MET activation generally occurs as a late event, in consequence of transcriptional upregulation driven by unfavorable microenvironmental conditions, such as hypoxia or ionizing radiation. Notably, growing evidence sustains that MET activation may collaborate in maintaining tissue plasticity and the regenerative potential that characterizes IPF [90]. We and others have already reported that both myofibroblasts and epithelial cells of fibroblast foci (FF) in IPF harbor MET in its activated form. Thus, although deregulation of the MET signaling cascade is clearly implicated in the development of IPF, some issues require clarification. IPF resembles cancer in many MET-associated behaviors, such as invasive phenotype and pro-coagulant status [91,92]. During the EMT process, cells acquire myofibroblast markers (like SMA) and fibroblast markers (like FSP1) while losing epithelial markers (e.g., E-cadherin, zonula occudents-1) [93,94]. Myofibroblast death is a normal process during wound healing; however, if these cells are unable to undergo apoptosis, myofibroblast accumulation and continuous extracellular matrix formation may occur (ECM) [95]. MicroRNA arrays have been used to observe some intriguing things in IPF lungs. Short RNA molecules called microRNAs (miRNAs) have a nucleotide count of about 22 and are used to either block or stimulate the degradation of target mRNAs. Myofibroblasts from IPF patients’ lungs have been shown to express the microRNA family miR-21 aberrantly, but not those from healthy controls. It has been demonstrated that miR-21 functions as an oncogene, delaying the senescence of myofibroblasts and promoting their proliferation and differentiation. While miR-21 is overexpressed, miR-29, which suppresses fibrogenic proteins, is underexpressed; this could lead to increased synthesis of connective tissue in IPF lung fibroblasts [96]. In IPF lungs, there is a differential expression of other microRNAs that have either profibrotic function (miR-145, miR-154, miR-199a-5p) or antifibrotic effects (miR-326, miR-17~92). When considered collectively, these data imply that fibroblasts from IPF patients may have a distinct phenotype characterized by uncontrollably high cell proliferation and the synthesis of connective tissue proteins due to abnormal post-transcriptional gene regulation [97,98,99,100,101,102].

### 4.9. Collagen Deposition

Myofibroblasts produce excess collagen in idiopathic pulmonary fibrosis (IPF), which is then deposited in an unorganized fashion within the extracellular matrix. Collagen type I is the most common form in areas of mature fibrosis, whereas collagen type III is more common in areas of early fibrosis. Techniques such as immunostaining and in situ hybridization have revealed a higher concentration of fibroblasts that synthesize collagen in the lungs of IPF patients. These fibroblasts, which are grouped together, create the recognizable fibrotic foci that are observed in the subepithelial regions of wounded lung tissue. These foci are on the airspace side of the wounded alveoli, as seen by the remnant patches of basal lamina around them. Patients with IPF have fibroblasts that synthesize collagen in their lungs, but this is not the case in normal individuals [103]. Regarding the cause of increased collagen deposition in pulmonary fibrosis, contradictory information has been found. Lung biopsies from IPF patients have shown increased production of collagen and a greater number of collagen-synthesizing cells [104]. In contrast to control cell lines, fibroblasts isolated from the lungs of patients with IPF and systemic sclerosis were found to produce a comparable amount of collagen, albeit at a lower rate of collagen breakdown. Theoretically, decreased collagenolytic activity could be a factor in excess collagen deposition in IPF in addition to increased collagen production and deposition [105].

## 5. Discussion

Although IPF remains an orphan disease and not all the pathologic mechanisms involved in its onset have been fully clarified, significant progress has been made. Overall, the paradigmatic frame now encompasses the complex interplay between injured epithelial and fibroblast cells under a process recalling EMT and the acquisition of an undifferentiated phenotype, which characterizes myofibroblasts. There is thus a strong rationale to develop novel drugs able to impair tissue plasticity and regeneration. The last decade saw some steps forward in IPF treatment; after abandoning more empiric and wide-spectrum strategies, nowadays the new therapeutic rationale is based on finding new kinds of more specific targets tailored to the pathogenetic process of the disease. The main goal of current research efforts is to keep this therapy-tailoring process evolving, thus allowing future clinical practice to shift from a disease-slowing approach to a disease-controlling one. In this perspective, the most promising approaches are those exploiting mesenchymal stromal cells. Increasing knowledge in this context derives from the recent experience of COVID-19 treatment [106] and lung transplantation [107].

### 5.1. Current Therapeutic Approaches

A decade ago, IPF’s main therapeutic approach was based on a combination of prednisone, NAC, and azathioprine [108], until the PANTHER-IPF trial showed in 2012 an increased risk of death, hospitalization, and therapy-related severe adverse events following this kind of protocol [109]. Ulterior studies investigated the possible use of other types of immunosuppressants such as micofenolate and cyclophosphamide, but showed no benefit [110,111]. Thalidomide was evaluated in consideration of its anti-inflammatory properties and showed a moderate efficacy in the reduction of lung collagen deposition in bleomycin-induced IPF trials, mitigating the upregulation of TGF-b and IL-6, which play an essential role in the profibrotic pathway and in the epithelial–mesenchymal transition [112,113]. Current clinical practice allows the introduction of an anti-fibrosing therapy in patients satisfying criteria for progressive pulmonary fibrosis (PPF), defined as the presence of at least two of the following in the last year: (i) worsening respiratory symptoms; (ii) functional decline; (iii) radiological progression. The currently approved drugs are nintedanib and pirfenidone. 

#### 5.1.1. Nintedanib

Nintedanib inhibits tyrosine kinases in many growth factors, such as platelet-derived (PDGFR), fibroblast (FGFR), and vascular-endothelial (VEGF). Specifically, it competitively binds triphosphate–adenosine binding sites, blocking intracellular signalling pathways related to fibrotic tissue remodelling in ILD pathogenesis [114]. Data from in vitro studies have shown that nintedanib inhibits fibroblast proliferation, migration, and differentiation and the secretion of ECM [115,116]. Its approval against IPF was evaluated in randomized, double-blind, placebo-controlled, multinational, phase 3 INPULSIS-1 and INPULSIS-2 trials. Both trials showed a reduction in FVC decline at week 52 of treatment with nintedanib, showing a mean difference of 125.3 mL/year between the drug and placebo groups in INPULSIS-1 and 207.3 mL/year in INPULSIS-2. In INPULSIS-2, there was also a significant between-group difference in the number of people having more than one acute exacerbation (3.6% vs. 9.6%). No between-group difference was shown in terms of death for all causes [117]. Nintedanib showed a higher proportion of gastrointestinal adverse events compared to placebo, namely, diarrhoea and nausea, along with elevation of liver enzymes (4.9% vs. 0.5%). Diarrhoea led to drug discontinuation in approximately 5% of patients. In both trials, the proportion of patients with serious adverse events was similar in the placebo and nintedanib groups. 

#### 5.1.2. Pirfenidone (PFD)

Pirfenidone can act on two different pathogenetic pathways, namely, on an anti-fibrosing and anti-inflammatory point of view. Firstly, it reduces collagen synthesis and fibroblast proliferation by inhibiting the production of many growth factors, mainly represented by TGF-b, but also fibroblast (FGF) and platelet-derived ones (PDGF), as shown in many studies evaluating its potential efficacy in slowing cardiac fibrosis [118,119]. Secondly, PFD modulates the inflammatory pathway by reducing the expression of many cytokines, such as IL-4 and IL-13 [120]. According to a recent study, PFD-mediated cytokine inhibition can also reduce B cell-mediated fibroblast activation and migration [121]. The first phase 3 clinical trial evaluating its employment in treating IPF was conducted in Japan in 2010 and involved 275 patients; in this study, pirfenidone showed an improvement in progression-free survival and a reduction in the decline of forced vital capacity after 52 weeks of treatment [122]. Two successive trials, CAPACITY and ASCEND, confirmed a reduction in clinical and functional decline compared to the placebo group, giving further support to the approval of pirfenidone in clinical practice; more specifically, ASCEND showed that in the pirfenidone group the number of patients with a significant decline in FVC (intended as >10% of predicted) was 49.9% lower than the placebo group at week 52. The mean decline in FVC from baseline was 428 mL in the placebo group and 235 mL in the pirfenidone group. Similarly, the number of patients showing a significant (>50 mt) decline in 6MWT distance from baseline to week 52 was 27.5% lower in pirfenidone than placebo [123]. In terms of all-cause mortality, the pirfenidone group showed a lower number of deaths compared to placebo, but this difference was not statistically significant. The main adverse events were gastrointestinal and skin-related but conducted to discontinuation of study only in few cases (14% of pirfenidone group). Elevation in transaminases occurred in 2.9% of the pirfenidone group versus 0.4% of the placebo group, but all cases were reversible and free of severe clinical complications. Interestingly, the INJOURNEY trial showed that treatment with nintedanib and add-on pirfenidone in IPF patients led to a good tolerability profile, supporting the need for further investigation of combination therapy’s efficacy [124].

#### 5.1.3. Not Only IPF

Although nintedanib and pirfenidone’s efficacy was first studied for IPF treatment only, successive studies tested their efficacy across a broad range of interstitial lung diseases; both drugs are nowadays used in current clinical practice to treat patients meeting criteria for progression of non-idiopathic interstitial fibrosing lung disease. In particular, nintedanib’s efficacy in this field was proven in many clinical trials [125], especially in systemic sclerosis (SScl)-related ILDs [126,127,128]. On the other hand, clinical trials investigating pirfenidone’s efficacy in non-IPF patients did not produce clear evidence supporting its clinical use, as many studies did not reach their primary outcome or were prematurely interrupted [129,130]; data are still lacking, and further investigation is needed. Nonetheless, PFD employment is being studied in rheumatoid arthritis-related ILDs, showing a reduction in FVC over time [131], while its efficacy in SScl-related ILD was not proven [132]. Moreover, a recent meta-analysis showed a similar efficacy between the two anti-fibrotic drugs in slowing disease progression in both IPF and non-IPF patients [133]. 

### 5.2. The Role of Mesenchymal Stromal Cells 

**Figure 3:**
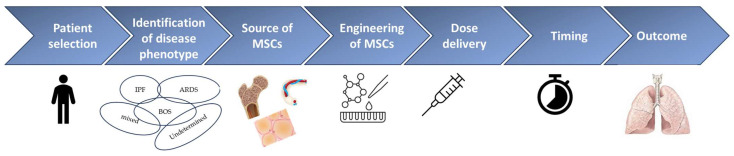
The journey to clinical development of advanced cell therapy. In this clinical context, a fully personalized approach requires proper selection of patients and of interstitial lung disease. Next, the definition of the optimal source of stem cells (in the case of MSCs, the most relevant are bone marrow, adipose tissue, and umbilical cord) and their advanced engineering will assure an adequate dose delivery. Definition of dose and time of administration represent the last steps towards the best clinical result.

Mesenchymal stromal cells (MSCs) are a population of non-hematopoietic multipotent cells with anti-inflammatory and immunomodulatory properties. They are present in numerous tissues, where they contribute to tissue renewal and homeostasis maintenance [107]. Resident lung MSCs are located in perivascular spaces, where they act as a source of cells for tissue repair after lung injury [134]. They exert their effects through cell-to-cell interactions and the production of a functional secretome composed of cytokines, indoleamine 2,3-dioxygenase, growth factors and other bioactive molecules, microRNAs, and extracellular vesicles acting in a paracrine manner. They reduce B cell proliferation and activation and the cytotoxic activity of natural killer cells, impair T cell proliferation, and reduce dendritic cell migration and maturation; they also promote immune tolerance through the polarization of naive T cells towards a T regulatory phenotype and the shift from an M1 to M2 macrophage phenotype [135,136,137,138]. MSCs promote lung epithelial and endothelial repair and contrast fibrogenesis by inhibiting the endothelial-to-mesenchymal transition and reducing collagen deposition [139,140,141,142,143]. Advanced cell therapy can be used as a therapeutic product and, in this context, must be manufactured according to good manufacturing practices (GMP) (Figure 3). MSCs can be isolated from bone marrow, adipose tissue, dental pulp, the umbilical cord, and the placenta, expanded in vitro and reinfused for therapeutic purposes. They have to respect strict quality criteria, defined by the International Society for Cell Therapy [144]: fusiform, fibroblast-like morphology, plastic adhesion in standard culture conditions, expression of typical stromal cell surface markers (CD 70, CD95, CD 105), lack of expression of hematopoietic markers (CD14 or CD11b, CD34, CD 45, CD79alpha or CD19), and ability to differentiate into different mesodermal cell lines, if appropriately stimulated. MSCs are immune-privileged, since they lack the expression of HLA-II and the costimulatory molecules/receptors CD80 and CD86, necessary for T cell response activation. This feature makes MSCs versatile because they can be used between HLA-mismatched individuals without inducing an immune reaction [122]. Their use has already been explored for several diseases such as cardiovascular disease [145], sepsis [146], neurological disease [147], autoimmune disorders [148], and graft-versus host disease [149]. In recent times, they have been investigated for the treatment of acute respiratory distress syndrome (ARDS) [134], especially *SARS-CoV-2*-related ARDS, and in lung transplantation [150]. As described above, IPF is a pathology of senescence characterized by an aberrant restorative response to repeated lung injuries, mitochondrial dysfunction, genomic instability, telomere shortening, and stem cell exhaustion. Due to their regenerative properties, MSCs appear conceptually to be a promising therapy able to promote tissue repair and disrupt the fibrotic process of IPF. Several pre-clinical studies, conducted especially on bleomycin-induced lung fibrosis murine models, investigated the role of MSCs from different sources administered with a dosage ranging between 0.1 × 10^6^ and 4 × 10^6^ cells. Most studies reported a decrease in lung collagen deposition, a decrease in the Ashcroft score, an improvement in histopathology, and a reduction in TGF beta levels, with conflicting results on other cytokines [151]. In one study, human adipose-derived MSCs showed superiority to pirfenidone in terms of survival in bleomycin-induced fibrosis mice and led to reductions in IL-2, IL-1b, TNF-α, TGFβ, and MMP levels, resulting in less deposition of extracellular matrix [152]. MSCs from different sources exert different actions both in vitro and in vivo. Bone marrow-derived MSCs (BM-MSCs) are the most accessible and commonly used cell types in lung diseases [153,154]. In vitro BM-MSCs have demonstrated superiority in promoting the shift towards the tolerogenic phenotype of macrophages [138], the reduction in CD4 and CD8 T cell expression [155], and the suppression of peripheral blood mononuclear cells levels compared with adipose tissue- or placenta-derived MSCs [156]. In vivo, as demonstrated by Periera Simon et al., who compared the effects exerted by MSCs on mice with bleomycin (BLM)-induced lung fibrosis according to their source, all types of MSCs were able to reduce the Aschroft score after 10 days of treatment; further, all MSCs, except placental ones, decreased hydroxyproline and αv-integrin and TNF-α mRNA levels. Instead, only adipose- and chorionic-derived MSCs reduced AKT and MMP-2 activation, whereas adipose MSCs increased Cav-1 levels; finally, only adipose-derived MSCs attenuated dysregulation of miR-29 and miR-199 [157]. However, in the translation of pre-clinical results to humans, concern remains about the possibility of MSCs to promote fibrosis in the context of the pro-fibrotic microenvironment of IPF. Moreover, the heterogeneity of tissue source, dose, timing and frequency of administration, and delivery of MSCs makes it difficult make comparisons between human studies. Several clinical trials of MSCs in patients with IPF have shown good safety profiles. In the first phase 1b trial, conducted in 2013- 14, mild-to-moderate IPF patients received three endobronchial infusions of autologous adipose-derived stromal cells at a dosage of 0.5 million cells per kilogram of body weight per infusion. The treatment was well-tolerated (the main adverse event was transient fever in 50% of cases) and no death, acute exacerbation, or ectopic tissue formation were reported after 2 years of follow-up [158]. Based on those results, five other phase 1 trials on IPF have been conducted. The AETHER trial demonstrated the safety of a single IV infusion of 20, 100, or 200 × 10^6^ human bone marrow-derived mesenchymal stem cells in nine patients with IPF [159]. In Chambers’ cohort, both schedules of 1 × 10^6^ and 2 × 10^6^ per kilogram patient weight allogenic, placenta-derived MSCs were well-tolerated in eight patients with IPF [160]. Later, the same group administered MSCs (in this case of bone marrow derivation, at a dose of 2 × 10^6^ per kilogram of patient weight) in ten patients with advanced chronic lung allograft dysfunction (CLAD) after lung transplantation, with comparable results in terms of safety [161], confirming the potential use of MSCs in different models of lung fibrosis. The group of Averyanov et al. compared MSC treatment (four treatments of 2 × 10^8^ bone marrow-derived, allogenic cells every 3 months) with placebo in patients with IPF, showing a stabilization of DLCO and FVC up to 26 weeks of treatment, an increase in the 6MWDT from week 13, and an increase in FVC by week 39 in the treatment group compared with the placebo group [162]. The results of the remaining clinical trials on the use of MSCs in IPF have not yet been published (NCT02135380, NCT01919827, website at https://clinicaltrials.gov/, accessed on 1 December 2023). MSCs have been demonstrated to be able to home to the lungs, differentiating in epithelial cells and reducing inflammation and extracellular matrix deposition in murine models of lung fibrosis [163]. They migrate in response to damage of epithelial alveolar cells through the release of chemotactic factors [164], such as the chemokine SDF-1 interacting with its ligand CXCR-4 [165] and the chemokine CXCL8 (interleukin-8) interacting with CXCR-2 [150]. The delivery mode is crucial for exploiting their therapeutic activity. Two ways are used to administer MSCs: systemic (intravenous) and local (intratracheal) delivery. The intravenous administration allows the exploitation of the first passage effect, that is, the phenomenon in which drugs passing through lungs remain trapped within the pulmonary vasculature, where they exert their effect. Moreover, this administration is easy and often repeatable. In contrast, the intratracheal delivery prolongs the cell half-life and reduces systemic side effects [138]. In IPF, a potential route of delivery of MSCs is the aerosolic one, as already tested on rabbits [166]. MSCs act in a dose-dependent manner [167]. The optimal dosage of administration is still not clear, but a too-low number of cells results in therapeutic ineffectiveness, while a too-high number of cells increases vascular resistance and may cause pulmonary embolism [168]. In addition, some strategies of genetic engineering may be applied to increase the efficacy of MSCs. For example, BM-MSCs modified with addition of BMP-7 have been demonstrated to be able to reduce the epithelial–mesenchymal transition in murine models [169]. Pretreatment of MSCs with oncostatin M prolongs their survival and upregulates HGF secretion [170]. The addition of G-CSF increases homing to lungs and expression of CXCR4, attenuating lung fibrosis [171], while pretreatment with N-acetylcysteine enhances intracellular glutathione levels and antioxidant activity [172]. Beyond MSCs, alveolar type II (AT II) cells can also act as progenitor stem cells, able to transdifferentiate into alveolar type I (AT I) cells, thus promoting lung regeneration [173,174]. The process of transdifferentiation is very active during lung embryogenesis but occurs at low rate in physiological conditions in adults; however, in case of lung injury, it allows the restoration of tissue integrity [175]. The mechanism of lung regeneration mediated by ATII cell transdifferentiation is not well-characterized, but it seems to involve the Notch and Wnt signalling pathways at the molecular level. The passage from AT II to AT I cells passes through a phase where cells express KRT8 (an intermediate alveolar epithelial cell marker) and both markers of AT II (SPC) and AT I cells (RAGE) [176]. An impairment of transdifferentiation from AT II to AT I cells contributes to lung fibrosis [177,178]. On this basis, in experimental models of mice with bleomycine-induced lung fibrosis, intratracheal transplantation of ATII cells has reduced collagen deposition and restored levels of SPC surfactant protein [179,180]. In humans, after the good results in terms of safety of the first study regarding ATII-cell intratracheal transplantation on 16 patients with IPF [181], the first phase 1 clinical trial on intravenous delivery of lung spheroid stem cells derived from autologous trans-bronchial pulmonary biopsy specimens is ongoing. The number of patients expected to be recruited is 24, divided into three arms (100 million cells vs. 20 million cells vs. placebo) (NCT04262167, website at https://clinicaltrials.gov/, accessed on 1 December 2023).

In recent years, some investigators have conducted meta-analysis studies to identify potential variables affecting cellular therapies based on MSCs. Donor variance, ex vivo expansion and senescence, immunogenicity, and cryopreservation are among the main factors that can compromise the effectiveness of MSC transplants. The immunoregulatory properties of MSCs may exhibit significant inter-donor variability. Interferon-gamma-induced IDO (indoleamine 2,3-dioxygenase) upregulation may be used as a marker of immunosuppression activity, and the authors should consider addressing this issue. The importance of senescence in the failure of stem cell-based trials has been emphasized as well [182,183]. 

### 5.3. Senolytics and Other Drugs under Investigation

Another relevant aspect is the type of fibrotic factors that could be released by senescent MSCs. Lung senescence is characterized by growth arrest, resistance to apoptosis, stem cell exhaustion, and the production of pro-inflammatory and pro-fibrotic senescence-associated secretory phenotype (SASP) factors in response to cellular stress [184,185]. Studying and analyzing SASP is of great interest, since senescence processes can spread to other elements with consequent clinical impact. Indeed, senescence is a dynamic program. A recent meta-analysis pointed out that: (i) IGF and its binding proteins are relevant transducers involved in the pathway; (ii) runt-related transcription factor 1 (RUNX1) and UCH deubiquitination [186], which are known to regulate proteasome activity, are overexpressed in the early phases of the program; (iii) inflammatory proteins and interleukins (such as IL-1, -4, -12, -13, and NF-kb) are abundant in the late stages; (iv) a shift from carbohydrate metabolism to glycolysis is observed; (v) senescent fibroblasts induced by oncogene-induced senescence (OIS) [187] display a different phenotype from other senescent cells [188].

Cellular senescence is crucial for cell replacement and tissue homeostasis. In IPF, in response to repeated injuries, there is a derangement of the senescent process with the production of a repertoire of cytokines, chemokines, proteases, and extracellular matrix components by senescent fibroblasts and epithelial cells resulting in irreversible scarring, parenchymal distortion, and lung function impairment [189]. In fact, markers of senescence such as β-galactosidase activity (SA-β-gal), p16, and p21 have been found in fibroblasts and epithelial cells in lung tissue biopsies [168].

Two types of strategies have been developed to counter SASP: senolytic and senomorphic drugs. The first ones block anti-apoptotic pathways and allow the removal of senescent cells [190]. Senomorphic agents suppress SASP and limit the proliferation of senescent cells without destroying them [191]. They are under investigation for several chronic diseases typical of aging, such as Alzheimer’s disease [192].

In 2019, the first pilot study in humans was conducted to study the effect of a combination of the senolytic drugs dasatinib (100 mg/die) and quercetin (1250 mg/die), given three days/week for a duration of three weeks, in 14 IPF patients. Non-serious adverse effects were reported; pulmonary function tests, laboratory tests, and frailty indexes were unchanged [193]. 

In a very recent phase 1 randomized, placebo-controlled pilot trial by Nambiar et al., twelve IPF patients received dasatinib (100 mg/die) and quercetin (1250 mg/die) three consecutive days per week for a total of 108 doses. Treatment was promising in terms of feasibility and safety [194]. Dasatinib is an oral tyrosine kinase inhibitor, while quercetin is a flavonoid with antioxidant properties able to induce autophagy through proteasome activation and to promote cell apoptosis through FAS ligand and TNF [195]. The two drugs probably act synergistically. In 31 patients with systemic sclerosis-associated ILD, dasatinib alone (100 mg daily for 6 months) was not effective [196].

The mechanism of action is still not completely known, but it is based on an induction of apoptosis in senescent cells [171], which entails a very low specificity in cellular target, so that many studies suggest caution in their employment [197]; notably, targeting the NOX4 enzyme has been shown to induce senescent myofibroblast apoptosis [198]. In this regard, a randomized, double-blind, placebo-controlled phase 2 clinical trial of GKT137831 in IPF is ongoing and estimated to terminate in April 2024 (NCT03865927).

#### 5.3.1. Pentraxins

Pentraxins are a family of liver-derived acute-phase proteins involved in injured tissue remodeling and wound healing; specifically, they inhibit TGF-b production, resulting in a reduction in monocyte differentiation into profibrotic macrophages and fibrocytes. Pentraxin 2 (PTX2 or serum amyloid P) is currently under investigation, as it might help reduce alveolar remodeling and clinical–functional worsening. As asserted in previous studies [199], circulating fibrocyte levels correlate with the abundance of fibroblastic foci in IPF patients, predicting an average lifespan of 7.5 months when above 5% of total blood leukocytes, compared to 27 months when below 5%. A first in-human trial of recombinant pentraxin-2 employment was performed in 2013, suggesting a reduction in fibrocytes in pulmonary fibrosis patients compared to healthy volunteers [200]. In 2016, an RCT suggested forced vital capacity and 6min walk test trends towards improvement in IPF patients after intravenous treatment on days 1, 3, 5, 8, and 15 [201]. In 2018, a preliminary study comparing human PTX-2 to placebo showed a slower decline in DLCO and 6MWT distance over 28 weeks in IPF [35].

Long-term evaluation of PTX-2 treatment was assessed in two more recent trials: the first one administered intravenous infusions on days 1, 3, and 5 in the first week of each cycle followed by one infusion of 10 mg/kg every 4 weeks, accounting for a total of 28 weeks of treatment. The results at week 52 showed an FVC decline of −3.6% per year and a decline in 6 min walking distance of −10.5 m per year compared to the placebo group. 

The latter study showed that treatment prolongation up to 128 weeks was well-tolerated; there were no significant differences in terms of FVC and 6MWT distance between people starting versus continuing the medication. The limited efficacy data were interpreted as a possible suggestion of a trend toward treatment [202]. 

#### 5.3.2. Pamrevlumab

Pamrevlumab is a monoclonal antibody targeting connective tissue growth factor (CTGF) currently under evaluation for treatment of IPF and non-resectable pancreatic cancer [203]. The PRAISE trial showed a similar effectiveness compared to pirfenidone and nintedanib, with a mean change from baseline to week 48 in FVC of −2.9% of the predicted value in the pamrevlumab group versus −7.2% in the placebo group, also maintaining a favorable safety profile [204]. The ZEPHYRUS trial (NCT03955146) is ongoing and will evaluate Ppamrevlumab’s efficacy and tolerability. 

A large number of molecules are now under clinical investigation in both phase 2 and 3 trials (Table 1), whereas more than 27 phase 1 trials are evaluating investigative treatments for IPF, many in the early phase or not yet recruiting. According to Global Data, phase 1 drugs for IPF have a 66% chance of moving on to phase 2 (website at https://www.globaldata.com/store/report, accessed on 1 December 2023).

## 6. Conclusions

A number of molecular mechanisms involved in organ regeneration and displaying protective functions appear to be activated in IPF. Thus, in IPF, epithelial cells are aberrantly activated in consequence to chronic wound edges and exploit growth signals to enhance cellular division and repopulation of the injured areas. A deeper understanding of the complex interplay between lung epithelial and endothelial cells and lung fibroblasts within the context of the senescent milieu that reproduces IPF plasticity is mandatory to identify novel targets that simultaneously block multiple pathways leading to pulmonary fibrosis. Most interest from the experimental and pre-clinical perspectives is now addressed to identifying the precise mechanisms of lung regeneration to discover new druggable targets for the development of regenerative therapies and to associate both strategies of fibrotic process disruption and alveolar repair and regeneration.

## Figures and Tables

**Table 1:** Investigative IPF treatments in human trials. It should be noted that the primary endpoint in most of the phase 2 trials is a change in ppFVC capacity from baseline to week 24.

Phase 3 Trial	
Molecule	Study Design
**Anlotinib**	A phase 2 and 3 trial in China is evaluating 1-year outcomes of once-daily oral anlotinib for treatment of IPF/progressive fibrosis–interstitial lung disease (PF-ILDS) (NCT05828953). Anlotinib is a tyrosine kinase inhibitor (TKI) that targets four factors: vascular endothelial growth factor receptor (VEGR), fibroblast growth factor receptor (FGFR), platelet-derived growth factor receptors (PDGFR), and c-kit. It is approved in China as a third-line therapy for non-small-cell lung cancer (NSCLC).
**BI 101550**	Enrollment in the FIBRONEER-IPF trial commenced in autumn 2022 (NCT05321069), with completion scheduled for 2024. BI 1015550 is an oral phosphodiesterase 4B (PDE4B) inhibitor. FIBRONEER-ILD is a separate phase 3 trial in fibrosing idiopathic lung disease (NCT05321082). In both trials, the primary endpoint is the absolute change from baseline in FVC at week 52.
**BMS-986278**	Results of a phase 2 trial showed that twice-daily treatment with oral BMS-986278 60 mg over 26 weeks reduced the rate of decline in ppFVC by 69% vs. placebo. The phase 3 ALOFT trial has been approved but has not yet started recruiting patients (NCT06003426). BMS-986278 is a lysophosphatidic acid receptor 1 (LPA1) antagonist.
**Lansoprazole**	Commonly used to treat and prevent gastrointestinal problems like stomach ulcers and esophagitis, this oral proton pump inhibitor (PPI) is the focus of a trial in the United Kingdom evaluating if PPIs can slow the progression of IPF (NCT04965298).
**N-acetylcysteine (NAC)**	The PRECISIONS trial is evaluating the effect of NAC plus standard-of care treatment in IPF patients who have the TOLLIP rs3750910 TT genotype (NCT04300920). Participants receive 600 mg NAC orally or matched placebo three times daily for 24 months. Trial completion is scheduled for 2025.
**Treprostinil**	Already approved to treat pulmonary arterial hypertension and pulmonary hypertension associated with interstitial lung disease, inhaled treprostinil is the subject of the TETON 1 and 2 trials evaluating its impact on ppFVC after 52 weeks of treatment (NCT04708782, NCT05255991).
**Phase 2 Trial**	
**Molecule**	**Study Design**
**Bexotegrast (PLN-74809)**	(PLN-74809) is an oral, small-molecule, dual-selective inhibitor of alphav/beta6 and alphav/beta1 (NCT04396756).
**BBT-877**	Described as a potent autotaxin (ATX) inhibitor, BBT-877 demonstrated its ability to inhibit lysophosphatidic acid (LPA) production by as much as 90% (NCT05483907).
**CC-90001**	It is an oral, once-daily c-Jun N-terminal kinase inhibitor. JNKs have been implicated in the underlying mechanisms of fibrosis, including epithelial cell death, inflammation and polarization of profibrotic macrophages, fibroblast activation, and collagen production (NCT03142191).
**C21**	C21 targets the underlying fibrosis in IPF by stimulating the protective arm of the renin–angiotensin system. It also has an upstream effect by promoting alveolar repair, by which it can reduce fibrosis formation, stabilize disease, and increase lung capacity (NCT04533022).
**CSL312 (garadacimab)**	It is a humanized anti-FXIIa monoclonal antibody administrated intravenously (NCT05130970).
**Cudetaxestat**	It is a noncompetitive autotaxin inhibitor (NCT05373914).
**Bersiposocin/DWN12088**	Bersiposocin/DWN12088, an inhibitor of prolyl-tRNA synthetase 1 (PARS1), is suspected to control the pathologic accumulation of collagen containing high amounts of proline in fibrotic diseases (NCT05389215).
**ENV-101**	ENV-101 is a small-molecule inhibitor of the Hedgehog (Hh) signaling pathway, which plays a key role in IPF. This agent was originally developed to target Hh-driven cancers (NCT04968574).
**GKT137831 (setanaxib)**	It inhibits nicotinamide adenine dinucleotide phosphate (NADPH) oxidase (NOX) isoforms (NCT03865927).
**HZN-825**	HZN-825 is a lysophosphatidic acid receptor 1 (LPAR1) antagonist (NCT05032066).
**Ifetroban**	It is a potent and selective thromboxane–prostanoid receptor(TPr) antagonist, exhibits a high affinity for TPr in platelets, vascular and airway smooth muscle, and fibroblasts, and lacks agonistic activity (NCT05571059).
**INS018_055**	It is a small-molecule, oral antifibrotic candidate notable for being the first entirely AI-generated drug to enter phase 2 trials. Trial enrollment started in October (NCT05975983,NCT05983920).
**Jaktinib dihydrochloride mono- hydrate**	It is an oral JAK1, JAK2, and JAK3 inhibitor (NCT04312594).
**Leramistat**	It is an anti-tumor necrosis factor (TNF) agent (NCT05951296).
**LTP001**	It is an oral, selectively deuterated form of pirfenidone designed to retain the antifibrotic and anti-inflammatory activity of pirfenidone with a differentiated pharmacokinetic profile (NCT05497284, NCT05321420).
**ME-015 (suplatast tosilate)**	It aims to stabilize ion channels in the neuronal endings in the lungs that mediate IPF-related cough (NCT05983471).
**Nalbuphine**	It is a small-molecule, dual-mechanism treatment for chronic cough in IPF and acts as both a mu opioid receptor antagonist and a kappa opioid receptor agonist (NCT05964335). The CANAL trial, completed last year, is evaluating an extended-release formulation (NCT04030026).
**NP-120 (ifenprodil)**	It is a small-molecule N-methyl-D-aspartate (NMDA) receptor antagonist that specifically targets the NMDA-type subunit 2B (GluN2B) (NCT04318704).
**Orvepitant**	It is a selective antagonist for the NK1 receptor and is being evaluated to treat IPF-related cough (NCT05815089).
**RXC007 (zelasudil)**	It is a coiled-coil-containing protein kinase 2 (ROCK2) selective inhibitor and was granted FDA orphan drug designation in August 2023 (NCT05570058).
**Saracatinib**	It is a selective Src kinase inhibitor originally developed for oncological indications (NCT04598919).
**SHR-1906**	For intravenous treatment, it inhibits the binding of a target protein to a variety of cytokines and growth factors, affects downstream signaling pathways, and reduces cell proliferation and migration (NCT05722964).
**TTI-101**	It is an oral, small-molecule inhibitor of signal transducer and activator of transcription (STAT3), which has been found to accumulate in the lungs of IPF patients (NCT05671835).
**VAY736 (lanalumab)**	It is a BAFF-R inhibitor (NCT03287414).
**Vixarelimab**	It is a human monoclonal oncastatin M receptor beta antibody (NCT05785624).

The bold is the molecule name.
